# Monitoring of adherence to headache treatments by means of hair analysis

**DOI:** 10.1007/s00228-016-2163-5

**Published:** 2016-11-20

**Authors:** Anna Ferrari, Manuela Licata, Cecilia Rustichelli, Carlo Baraldi, Daniele Vandelli, Filippo Marchesi, Federica Palazzoli, Patrizia Verri, Enrico Silingardi

**Affiliations:** 1 Unit of Medical Toxicology, Headache Centre and Drug Abuse; Department of Diagnostic, Clinical and Public Health Medicine, University of Modena and Reggio Emilia, Policlinico, Via del Pozzo, 71, 41124 Modena, Italy; 2 Forensic Toxicology Laboratory, Department of Diagnostic Medicine, Department of Diagnostic, Clinical and Public Health Medicine, University of Modena and Reggio Emilia, Via del Pozzo, 71, 41124 Modena, Italy; 3 Department of Life Sciences, University of Modena and Reggio Emilia, via G. Campi, 103, 41125 Modena, Italy; 4 Unit of Forensic Medicine, Department of Diagnostic, Clinical and Public Health Medicine, University of Modena and Reggio Emilia, Via del Pozzo, 71, 41124 Modena, Italy

**Keywords:** Hair analysis, Headache, Adherence, Monitoring, Drug treatment, Prophylaxis

## Abstract

**Purpose:**

The aim of this study was to evaluate the potential of hair analysis to monitor medication adherence in headache patients undergoing chronic therapy. For this purpose, the following parameters were analyzed: the detection rate of 23 therapeutic drugs in headache patients’ hair, the degree of agreement between the self-reported drug and the drug found in hair, and whether the levels found in hair reflected the drug intake reported by the patients.

**Methods:**

The study included 93 patients suffering from primary headaches declaring their daily intake of at least one of the following drugs during the 3 months before the hair sampling: alprazolam, amitriptyline, citalopram, clomipramine, clonazepam, delorazepam, diazepam, duloxetine, fluoxetine, flurazepam, levomepromazine, levosulpiride, lorazepam, lormetazepam, mirtazapine, paroxetine, quetiapine, sertraline, topiramate, trazodone, triazolam, venlafaxine, and zolpidem.

A detailed pharmacological history and a sample of hair were collected for each patient. Hair samples were analyzed by liquid chromatography-electrospray tandem mass spectrometry, using a previously developed method.

**Results:**

All 23 drugs were detected in the examined hair samples. The agreement between the self-reported drug and the drug found in hair was excellent for most analytes (*P* < 0.001, Cohen’s kappa); a statistically significant relationship (*P* < 0.05, linear regression analysis) between dose and hair level was found for amitriptyline, citalopram, delorazepam, duloxetine, lorazepam, and venlafaxine.

**Conclusions:**

Hair analysis proved to be a unique matrix to document chronic drug use in headache patients, and the level found for each individual drug can represent a reliable marker of adherence to pharmacological treatments.

**Electronic supplementary material:**

The online version of this article (doi:10.1007/s00228-016-2163-5) contains supplementary material, which is available to authorized users.

## Introduction

Primary headaches are recurrent chronic disorders and highly prevalent: for example, in Europe, 53% of adults suffer from headache [1]. These disorders are debilitating, reduce the quality of life, and have enormous social costs and no resolutive cure [2]. Pharmacological treatments are the main tools to manage them. When acute headache episodes are frequent, a prophylactic treatment is indicated. If prophylaxis is to be effective, drugs are required to be taken daily for 3–6 months, independently from the occurrence of the attacks [3]. Moreover, headache patients often suffer from psychiatric comorbidities that aggravate the headache and require prolonged pharmacological treatments [4]. Unfortunately, between 25 and 50% of headache patients are not compliant with their pharmacological treatment; this fact represents a critical problem, since non-adherent patients fail to benefit from the prescribed therapy [5]. The problem is compounded by the fact that no tools are available in clinical practice to monitor headache patients’ adherence to long term treatments.

In recent years, various analytical protocols have been developed for a sensitive determination of drugs and related metabolites in hair samples, especially in the forensic field. Hair analysis could therefore represent an important tool also for therapeutic drug monitoring [6]. In spite of the large number of potentially determinable xenobiotics, the reported procedures only focused on the analysis of few therapeutic drugs, in a limited number of forensic cases, mostly on autopsy samples of hair belonging to subjects with unknown therapy (doses and length of treatment) [7, 8]. As a matter of fact, hair analysis as a tool for therapeutic drug monitoring has not received much attention in the clinical field, despite its potential usefulness, due to the noninvasive sampling technique, the wide detection window, and the possibility to determine simultaneously various analytes in the same sample [9, 10].

The goal of this study was to evaluate the usefulness of hair analysis in documenting headache patients’ adherence to chronic treatments. We therefore analyzed the detection rate of 23 therapeutic drugs in headache patients’ hair, the degree of agreement between the self-reported drug and the type of drug found in hair, and whether the concentrations measured in hair reflected the drug doses that the patients had reported to have taken in the previous 3-month period.

## Patients and methods

### Patients

This study is part of a research project concerning the analysis of therapeutic drugs in the hair of 300 primary headache patients consecutively afferent to a headache center. Only patients with a minimum occipital scalp hair length of 4 cm were enrolled in the study; patients who did not understand both the purpose and the protocol of the study were excluded. The patients were enrolled and hair specimens were collected from October 1, 2013 to December 23, 2014.

In this study we included 93 primary headache patients (mean age ± SD: 48.13 ± 12.03 years; females 94 %) who had reported to be in daily treatment, for at least 3 months, for headache prophylaxis or for their psychiatric comorbidities, with at least one of the following 23 drugs: alprazolam, amitriptyline, citalopram, clomipramine, clonazepam, delorazepam, diazepam, duloxetine, fluoxetine, flurazepam, levomepromazine, levosulpiride, lorazepam, lormetazepam, mirtazapine, paroxetine, quetiapine, sertraline, topiramate, trazodone, triazolam, venlafaxine, and zolpidem. All demographic and diagnosis data are available as a supplementary table.

### Procedures

For each patient, demographic data, hair characteristics, headache diagnosis, and a detailed pharmacological history (type of the drug taken, daily dose, and treatment duration) relative to the previous 3 months were collected. Drug quantities for each drug were converted in mg taken in 3 months.

According to the international guidelines for hair analysis [11], a hair sample of at least 7 mm in diameter and 4 cm in length was taken from each patient’s nuchal area. From each hair sample we cut and analyzed a single section measuring 3 cm, proximal, i.e., near the scalp, to cover the previous 3 months.

The extraction and purification of the samples were performed according to Licata et al. [12]. After decontamination and trituration, hair samples (50 mg aliquot) were added with 2.0 mL methanol and 20 μL of the IS solution and sonicated overnight at 45 °C; after addition of the roQ dSPE QuEChERS sorbent kit (2.0 mL, 150 mg MgSO4, 50 mg PSA-Primary Secondary Amine, 50 mg endcapped C18, Phenomenex, Torrance, CA, USA), the tubes were vortexed and centrifuged. One milliliter of each purified supernatant was evaporated to dryness and reconstituted in 200 μL of the LC mobile phase. The purified hair extracts were then subjected to LC-MS/MS analysis (injection volume, 10 μL) on a Kinetex® Biphenyl column under the gradient elution conditions. The method had been developed by us and validated [12] according to the model proposed by the Scientific World Group for Forensic Toxicology in 2013, in standard practices for method validation in forensic toxicology [11]. Experimental details concerning LC-MS/MS analysis and method validation have been reported in a previous paper [12] and are briefly summarized as an online supplementary document.

A number was assigned to each authentic hair sample, and the laboratory made blind assessments.

Quantitative analyses of the drugs were performed on the basis of the calibration curves daily prepared and analyzed as reported [12].

### Data and statistical analysis

A descriptive analysis of all variables was conducted as far as the following aspects were concerned: demographic characteristics, headache diagnosis, and pharmacological history.

Statistical analysis was carried out by the StataIC 13 software. The continuous variables normally distributed were expressed as mean ± standard deviation. The agreement between the type of drug reported by the patient and the one detected in hair was evaluated by Cohen’s K coefficient. For the drugs taken by at least five patients, the relationship between the reported cumulative dose (mg/3 months) and the corresponding drug hair levels was calculated by univariate linear regression analysis. *P* < 0.05 was chosen as significant for all the tests.

## Results

All 23 drugs (Table 1) were successfully identified in the examined hair samples: 82% of the parent drug (19/23) was found in 100% of patients declaring their intake in the previous 3 months. Only few hair samples resulted negative for the self-reported drug: amitriptyline (five hair samples), delorazepam (one hair sample), paroxetine (two hair samples), and venlafaxine (one hair sample). The agreement between the type of drug reported by the patient and the one detected in hair, analyzed by Cohen’s kappa, was statistically significant for all drugs (*P* < 0.001) and excellent (>0.8) for most of them. Nine hair samples were also positive for not declared drugs: citalopram, three hair samples; clomipramine, one hair sample; mirtazapine, two hair samples; sertraline, one hair sample; and zolpidem, two hair samples.

**Table 1:** Hair analysis results and agreement between the self-reported drug and drug found in hair samples. The 93 enrolled patients self-reported a chronic treatment with at least one of the target drugs during the 3 months preceding the hair sampling; some patients reported the intake of more than one drug during the examined time period

Self-reported drug	Number of positive hair samples (%)	Number of negative hair samples (%)	Number of patients self-reporting the drug	Kappa*
Alprazolam	7 (100)	0	7	1.0000^a^
Amitriptyline	24 (83)	5 (17)	29	0.8649^a^
Citalopram	16 (100)	0	16	0.8946^a^
Clomipramine	3 (100)	0	3	0.8517^a^
Clonazepam	1 (100)	0	1	1.0000^a^
Delorazepam	22 (96)	1 (4)	23	0.9707^a^
Diazepam	3 (100)	0	3	1.0000^a^
Duloxetine	16 (100)	0	16	1.0000^a^
Fluoxetine	4 (100)	0	4	1.0000^a^
Flurazepam	1 (100)	0	1	1.0000^a^
Levomepromazine	1 (100)	0	1	1.0000^a^
Levosulpiride	4 (100)	0	4	1.0000^a^
Lorazepam	7 (100)	0	7	1.0000^a^
Lormetazepam	1 (100)	0	1	1.0000^a^
Mirtazapine	2 (100)	0	2	0.6568^b^
Paroxetine	4 (67)	2 (33)	6	0.7891^b^
Quetiapine	2 (100)	0	2	1.0000^a^
Sertraline	4 (100)	0	4	0.8833^a^
Topiramate	12 (100)	0	12	1.0000^a^
Trazodone	1 (100)	0	1	1.0000^a^
Triazolam	1 (100)	0	1	1.0000^a^
Venlafaxine	6 (86)	1 (14)	7	0.9173^a^
Zolpidem	2 (100)	0	2	0.6568^b^
Total	144 (94)	9 (6)	153	

*All *P* values <0.001

^a^Kappa values from 0.8 to 1 = excellent agreement

^b^Kappa values from 0.6 to 0.8 = good agreement

Cumulative doses for the same drug taken by patients during the examined period (Table 2) differed considerably, leading to high SD values for this variable and to even higher SD values for hair levels found.

**Table 2:** Cumulative doses self-reported by the 93 patients in the previous 3 months and hair concentrations for the 23 drugs

Self-reported drug (*n*)	Cumulative doses (mean ± SD, mg)	Hair concentrations (mean ± SD, pg/mg)	*r*	*P* value
Alprazolam (7)	41.82 ± 44.09	66.95 ± 65.70	0.5339	0.431
Amitriptyline (24)	1877.50 ± 2218.01	2798.10 ± 3391.70	0.7485	0.015*
Citalopram (16)	1760.63 ± 868.18	2563.88 ± 1906.90	1.6653	<0.001**
Clomipramine (3)	6956.70 ± 11,513.30	3730.00 ± 3516.37		
Clonazepam (1)	90.00	90.00		
Delorazepam (22)	235.86 ± 395.16	150.26 ± 330.10	0.2563	<0.000**
Diazepam (3)	110.00 ± 43.59	1033.33 ± 1280.80		
Duloxetine (16)	4224.40 ± 1608.80	8520.75 ± 7627.80	1.2657	<0.001**
Fluoxetine (4)	2700.00 ± 1800	20,578.25 ± 22,440.20		
Flurazepam (1)	1350	359.00		
Levomepromazine (1)	2250.00	1037.00		
Levosulpiride (4)	2163.00 ± 3101.09	254.75 ± 217.40		
Lorazepam (7)	241.07 ± 298.13	263.71 ± 299.30	0.9995	<0.001**
Lormetazepam (1)	180.00	1207.00		
Mirtazapine (2)	2700.00 ± 1909.19	586.50 ± 227.90		
Paroxetine (4)	1350.00 ± 492.95	804.83 ± 709.90		
Quetiapine (2)	506.30 ± 79.50	470.40 ± 508.00		
Sertraline (4)	5812.50 ± 3986.30	24,600.25 ± 28,087.20		
Topiramate (12)	4774.17 ± 5343.41	2414.17 ± 2028.90	0.2210	0.217
Trazodone (1)	900.00	6379.00		
Triazolam (1)	22.50	16.00		
Venlafaxine (6)	6750.00 ± 6161.88	1864.14 ± 2174.90	0.3054	0.012*
Zolpidem (2)	2475.00 ± 2863.78	1718.00 ± 1750.80		

Linear regression analysis for drugs taken by at least five patients

*r* slope value

*P < 0.05

**P < 0.01

**Fig. 1:**
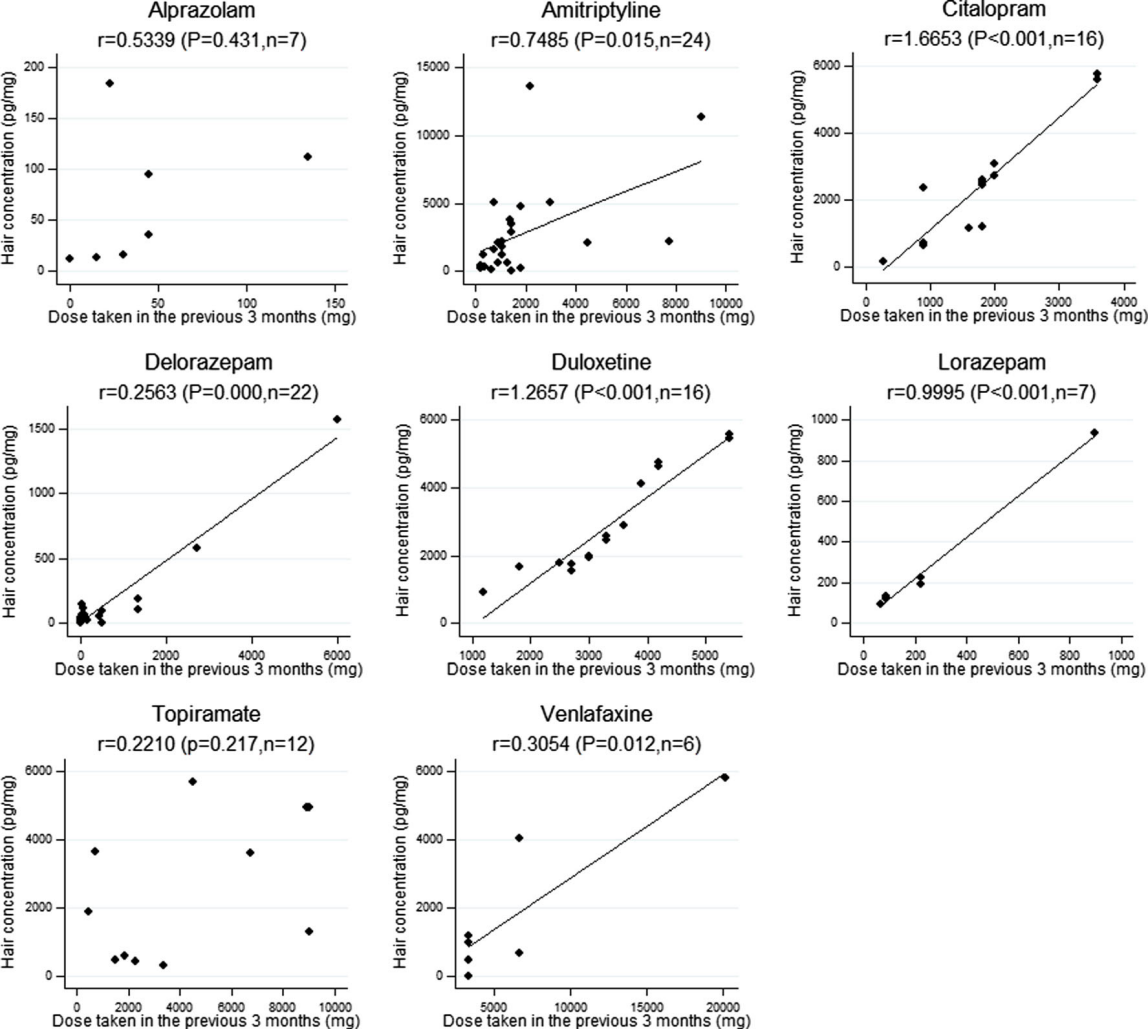
Relationship between cumulative dose of the drugs taken in the previous 3 months and their concentration in hair [*diamond* data points, *line* linear regression; the *fitted line* has only been reported when the regression coefficient was statistically significant (*P* < 0.05); *r* = slope value]

The relationship between cumulative doses and the levels found (Fig. 1), analyzed for the drugs positive in at least five patients, was statistically significant (*P* < 0.05, linear regression analysis) for amitriptyline, citalopram, delorazepam, duloxetine, lorazepam, and venlafaxine.

Considering the relationship between doses and hair levels and the calculated regression curve, the drug hair incorporation tendency was, in decreasing order, citalopram > duloxetine > lorazepam > amitriptyline > venlafaxine > delorazepam.

## Discussion

A fundamental requirement to employ hair analysis in drug monitoring is that the drugs are incorporated into the hair. Our results (Table 1) indicate that all 23 different therapeutic drugs daily taken at the doses indicated for headache prophylaxis or the treatment of psychiatric comorbidities could be determined in the keratin matrix. These drugs have in fact chemical characteristics, such as some degree of lipophilicity and basicity, enabling them to diffuse and be incorporated preferentially into the hair where the pH is acidic (about 4) [9]. Therefore, this matrix was a biological specimen suitable to analyze the concentration of these therapeutic drugs. In addition, the keratin matrix offers the possibility to determine simultaneously a large number of drugs in a unique sample [6], playing a crucial role in the case of polymedicated headache patients; in our series, 58 and 36% of the patients self-reported respectively two and three or more different types of drugs.

Medication adherence is critical to achieve the benefit of the cure and should be monitored [5]. To measure adherence to treatment, indirect methods, such as diaries or self-report, and direct ones, such as quantitative determination of the drugs in blood or urine, are available. The first overestimates the adherence, while the routine laboratory methods have a limited detection window, hours, at most days prior to sampling [13]. To verify adherence during prolonged treatments, several samples would need to be collected, causing excessive discomfort to patients. In addition, medications prescribed to headache patients are often not detected with traditional analytical methods.

Overall, there was a substantial concordance between the types of drugs self-reported by the patients and those detected by hair analysis. Indeed, 82% of the drugs were found in 100% of the samples of patients who had self-reported them. The agreement, analyzed by Cohen’s kappa, was statistically significant (*P* < 0.01) for all drugs and excellent (kappa >0.8) for most of them (87%). However, a certain degree of nonadherence was present: nine samples were negative for the self-reported drug and nine were positive for unreported drugs. We hypothesized that these results depended on patients’ mistakes or forgetfulness rather than on shortcomings of the previously validated analytical method [12].

The patients (Table 2) had taken different therapeutic doses, and the respective concentrations of the drugs in hair presented even greater interindividual variations, as shown by high standard deviation values. In order to interpret these data, the factors affecting drug concentrations in hair must be taken into account. Three factors are fundamental: (1) the individual characteristics of hair, such as color, cosmetic treatments, and growth rates ranging from 0.6 to 1.42 cm/month; (2) the interindividual variation in the kinetics of individual drugs, which affects circulating concentrations and therefore the penetration into growing hair from the bloodstream; (3) the chemical–physical characteristics of the drugs: increased lipophilicity and basicity and lower polarity and molecular weight facilitate the incorporation into the hair. Despite the mentioned variability, we found (Fig. 1) a statistically significant relationship between the doses taken and the corresponding concentrations in hair for most drugs self-reported by an adequate number of patients. On the basis of the regression line, citalopram and duloxetine, both very basic and lipophilic (the first has pKa 9.78 and logP 3.5 and the second pKa 9.7 and logP 4.72 [14]), were the best incorporated into the keratin matrix. Other studies, also carried out in the clinical setting, did not find a statistically significant relationship between daily dose and hair concentrations [15, 16]. However, these studies enrolled psychiatric patients or subjects in palliative care whose reliability on the medications taken was limited, as reported by the authors [15, 16]. In our research, the pharmacological history of each patient had been collected with extreme attention, although the study was not conducted in a setting of controlled drug administration.

The relationship between doses taken and measured hair concentrations was not statistically significant for topiramate, despite the number of patients who reported its intake. This result could depend on the considerable interindividual variations in the kinetics of this drug. In fact, the relationship between dose and plasma concentration is not linear [17] and serum concentrations of topiramate show considerable interindividual variations for the same administered dose [18].

Our study had some limits. The sample was not large, had a female prevalence, was intentionally heterogeneous for the types and doses of the drugs, and was also heterogeneous for hair color and evidence of cosmetic treatments, in order to assess the applicability and potentialities of hair analysis in various clinical situations. Moreover, it was possible to analyze the relationship between dose and concentration only for drugs taken by an adequate number of patients.

There is much research focused on the identification of drugs of abuse in the keratin matrix; however, that dealing with therapeutic drugs is rather scarce. We could not find published data on hair levels for duloxetine, levosulpiride, mirtazapine, paroxetine, and topiramate, and we could not therefore make comparisons. For the other drugs, measured concentrations were in agreement with literature, but the lack of information on the doses and duration of the treatments in published studies prevents precise comparisons [19–23].

By providing data on the doses and the related concentrations in clinical setting, our study could be a reference to attribute value to hair levels of the same drugs when the doses taken are unknown, for example in forensic toxicology.

## Conclusions

The results of our study indicated that hair analysis could be a valuable tool to document the adherence to prescribed therapeutic drugs in headache patients. The management of primary headaches is based solely on clinical history and patient reports [3]. In such situation, the concentration of drugs measured in hair represents a reliable marker of intake in the previous months. This information can help clinical and therapeutic decisions.

## Electronic supplementary material

ESM 1 (DOC 39 kb)

ESM 2 (DOC 73 kb)
