# Supplementary material for: Monitoring of adherence to headache treatments by means of hair analysis
Source: Eur J Clin Pharmacol. 2016 Nov 20;73(2):197–203. doi: 10.1007/s00228-016-2163-5 (PMC5226977; doi:10.1007/s00228-016-2163-5)
Supplement: Supplementary file 1 — (DOC 39 kb) [file 228_2016_2163_MOESM1_ESM.doc]

Demographic and treatments data

| **Variable** | **Number (%)** |
| --- | --- |
| Patients | 93 (100) |
| *Education* |  |
| Primary, secondary, high school | 69 (74) |
| University degree | 24 (26) |
| *Ethnia* |  |
| Caucasic | 91 (98) |
| African | 1 (1) |
| Asiatic | 1 (1) |
| *Setting* |  |
| Outpatients | 66 (71) |
| Inpatients | 27 (29) |
| *Diagnosis according to ICHD-3beta^*^* |  |
| Chronic migraine | 57 (61) |
| Migraine without aura | 18 (20) |
| Frequent episodic tension-type headache | 9 (10) |
| Chronic tension-type headache | 6 (6) |
| Migraine with and without aura | 3 (3) |
| *Hair colour* |  |
| Brown | 65 (70) |
| Black | 11 (12) |
| Grizzled | 9 (10) |
| Blond | 4 (4) |
| White | 4 (4) |
| *Chronic treatment* |  |
| One type of drug | 6 (6) |
| Two types of drugs | 54 (58) |
| Three or more type of drugs | 33 (36) |

* The International Classification of Headache Disorders, 3rd edition (beta version), 2013
