# Supplementary material for: Monitoring of adherence to headache treatments by means of hair analysis
Source: Eur J Clin Pharmacol. 2016 Nov 20;73(2):197–203. doi: 10.1007/s00228-016-2163-5 (PMC5226977; doi:10.1007/s00228-016-2163-5)
Supplement: Supplementary file 2 — (DOC 73 kb) [file 228_2016_2163_MOESM2_ESM.doc]

**LC-MS/MS analysis and method validation**

*LC-MS/MS parameters*

LC analyses were performed on a Kinetex® Biphenyl column (50 × 2.1 mm; 5.0 µm) preceded by a ULTRA Biphenyl Security Guard Cartridge (2.0 x 2.0 mm) (Phenomenex). Chromatographic elution was performed under gradient conditions with a mobile phase of (A) 1 mM aqueous ammonium formate (+ 0.1% formic acid) and (B) acetonitrile/methanol (70/30) (+ 1 mM ammonium formate, + 0.1% formic acid); total run time: 18 min. The flow-rate was 0.25 mL/min and the column temperature was 40°C. Tandem mass spectrometry was performed in ESI positive mode using scheduled multiple reaction monitoring (sMRM) technique, monitoring two transitions for each analyte and one transition for each Internal Standard (IS): Amitriptyline-D_3_, Citalopram-D_6_, Clozapine-D_4_, Desalkylflurazepam-D_4_, Fluoxetine-D_5_, α-Hydroxyalprazolam-D_5_ and Pinazepam.

The HPLC and MS/MS parameters were optimized by analyzing individual standard solutions and also extracts of blank hair spiked with the target analytes and the ISs.

*Validation*

Method validation was accomplished according to the Scientific Working Group of Forensic Toxicology (SGWTOX) standard practices for method validation in forensic toxicology [11].

The following parameters were evaluated: selectivity, calibration model, limit of detection (LOD), lower limit of quantitation (LLOQ), precision, accuracy, carry-over, matrix effects, recovery and dilution integrity.

*Selectivity*

Blank hair samples from different volunteers were processed and analyzed to investigate potential interferences; no significant peaks were present that could interfere with the analytes and the ISs, confirming the selectivity of the adopted procedure.

*Calibration curves, LOD and LLOQ*

Blank hair specimens were spiked with known amounts of the target analytes (10 calibration levels) and of the ISs (200.0 pg/mg hair); the internal standard with the closest retention time was assigned for each analyte to compensate for a possible matrix effect. The obtained calibration samples were processed and analyzed in triplicate (injection volume: 10.0 μL).

The relative response ratios of the quantifier transition (most intense transition) for each analyte to that of the assigned IS were plotted against the nominal analyte concentration to generate the standard curves by the method of the least squares. The obtained calibration curves were all linear in the range LLOQ-1500 pg/mg hair, with correlation coefficient (*R*^2^)≥ 0.990.

The LLOQ values were estimated by analysing blank samples fortified with decreasing analyte concentrations; the LOD values were calculated from the standard deviation of the *y*-intercept (s*_y_*) and the average slope (Avg*_m_*) as: LOD = 3.3 s*_y_*/Avg*_m_*. These values were then validated by analyzing blank hair samples fortified at the calculated LOD and LLOQ levels. The LLOQ values averaged 10.0−20.0 pg/mg hair, except for Fluoxetine (LLOQ: 50 pg/mg hair); the LOD values were in the range 2.0−10.0 pg/mg hair, except for Fluoxetine (LOD: 20 pg/mg hair).

*Precision and accuracy*

Method precision and accuracy were determined on samples spiked with the ISs (200.0 pg/mg hair) and with the analytes at three levels: low (20.0 pg/mg hair; for Fluoxetine: 50.0 pg/mg hair), medium (200.0 pg/mg hair) and high (1500.0 pg/mg hair); n=6 for each level.

The intra-batch precision and the inter-batch precision complied with the requirements reported in the guidelines [11], being the correspondent RSD% values < 20% for all analytes at the tested levels.

Accuracy was estimated by comparing the levels found in hair samples spiked after the sample processing with the nominal analyte concentration; the obtained values were always in the range ± 20%, which are within the acceptance criteria [11].

*Carry-over*

Blank hair samples analyzed after a high concentration sample (1500.0 pg/mg hair) showed a negligible carry-over effect, being the peak areas in the correspondent chromatogram <5% of the areas found for LLOQ.

*Matrix effect*

Matrix effect is the ratio of the response obtained from the analyte spiked into post-extracted blank matrix to the response for the neat standard. The values calculated at the three tested concentration levels (low, medium and high) were in the prescribed range (± 25%) [11], except for Levomepromazine, which exhibited a considerable enhancement effect (50.1%−82.1%); in this case the matrix effect has been compensated by the use of an adequate Internal Standard.

*Recovery*

Recovery values were calculated by comparing the concentrations found in samples spiked before the sample processing with those found in post-spiked samples at low, medium and high levels. Recovery values were > 84% for 14 analytes; further comments have been given in the published paper [12].

*Dilution integrity*

Blank hair samples, spiked at 40 times the high calibrator sample, were processed and diluted with extracts from blank hair samples; the obtained samples were analyzed against the calibration curves. The correspondent chromatograms showed adequate values for precision (RSD% < 20%) and accuracy (< ± 20%); therefore extracts of authentic hair samples with analyte concentrations exceeding the calibration range could be diluted to bring their concentration within the calibration range.
